# Esophageal Web in a Down Syndrome Infant—A Rare Case Report

**DOI:** 10.3390/children5010010

**Published:** 2018-01-11

**Authors:** Nirmala Thomas, Roy J. Mukkada, Muhammed Jasim Abdul Jalal, Nisha Narayanankutty

**Affiliations:** 1 Department of Pediatrics, VPS Lakeshore Hospital, 682040 Kochi, Kerala, India; drnirmala@lakeshorehospital.org; 2 Department of Gastromedicine, VPS Lakeshore Hospital, 682040 Kochi, Kerala, India; roymukkada@gmail.com; 3 Department of Family Medicine, VPS Lakeshore Hospital, 682040 Kochi, Kerala, India; nishanarayanan999@gmail.com

**Keywords:** trisomy 21, recurrent vomiting, aspiration pneumonia, esophageal web

## Abstract

We describe the rare case of an infant with trisomy 21 who presented with recurrent vomiting and aspiration pneumonia and a failure to thrive. Infants with Down’s syndrome have been known to have various problems in the gastrointestinal tract. In the esophagus, what have been described are dysmotility, gastroesophageal reflux and strictures. This infant on evaluation was found to have an esophageal web and simple endoscopic dilatation relieved the infant of her symptoms. No similar case has been reported in literature.

## 1. Introduction

In 1866, John Langdon Down described the physical manifestations of the disorder that would later bear his name [1]. Jerome Lejeune demonstrated its association with chromosome 21 in 1959 [1]. It is the most common chromosomal abnormality occurring in humans and it is caused by the presence a third copy of chromosome 21 (trisomy 21). It is associated with multisystem involvement with manifestations that grossly impact the quality of life of the child.

Esophageal web is a rare congenital anomaly of the esophagus. An esophageal web/ring is defined as a concentric, smooth, thin (3–5 mm) extension of normal esophageal tissue consisting of mostly mucosa and sub mucosa [2]. It typically causes partial obstruction in the middle to lower esophagus.

Here, we report a rare case of esophageal web in a one-year-old child with trisomy 21 born to second degree consanguineous parents.

## 2. Case Report

A one-year-old Omani female child presented to the emergency room with fever and severe respiratory distress. She was the sixth child of a second-degree consanguineous marriage and was found to have dysmorphic features at birth. Chromosomal analysis showed trisomy 21. Her cardiac echocardiogram was normal. Routine thyroid screens were normal. The baby had no respiratory problems in the initial newborn period. There was history of feeding difficulty since birth in the form of wet cough after feeding and the child had recurrent vomiting of unaltered food. The problems with feeding were accentuated when the semisolid feeds were commenced and she had multiple hospital admissions for vomiting and was documented to have had aspiration pneumonia on each of these admissions. She was treated symptomatically and discharged. She was presumed to have gastroesophageal reflux and no further evaluation was performed. She has been immunized to date and has global developmental delay with severe failure to thrive.

On examination, she was in severe respiratory distress with supra-sternal, intercostal, and subcostal recessions. She had noisy breathing with copious secretions and drooling. She was anicteric, acyanotic, but had moderate pallor. There was no clubbing, edema, or generalized lymphadenopathy.

The child was tachypneic with a respiratory rate of 60 breaths/min. Oxygen saturation was 78% in room air and 98% with 3 L oxygen/min. She was tachycardic with a heart rate of 120 beats/min. She was febrile with a temperature of 39 °C. Her blood pressure was 92/69 mm Hg.

The child had a weight of 5 kg, head circumference of 42 cm and length of 68 cm. Her developmental evaluation showed a developmental age of six months as opposed to a chronological age of one year for gross motor, speech and fine motor functions. Her weight was well below the first percentile for her age. She had good personal social interaction with gestures.

Complete physical examination showed typical morphological features of trisomy 21. Neurological examination showed decreased tone and normal deep tendon reflexes. All of her pulses were well felt. Heart sounds were normal. There were no murmurs and no features suggestive of cardiac failure. She had copious oral secretions and wet cough and was in respiratory distress. Breath sounds were equal bilaterally with coarse crepitations and rhonchi. Abdomen was soft and non-tender without organomegaly.

**Figure 1:**
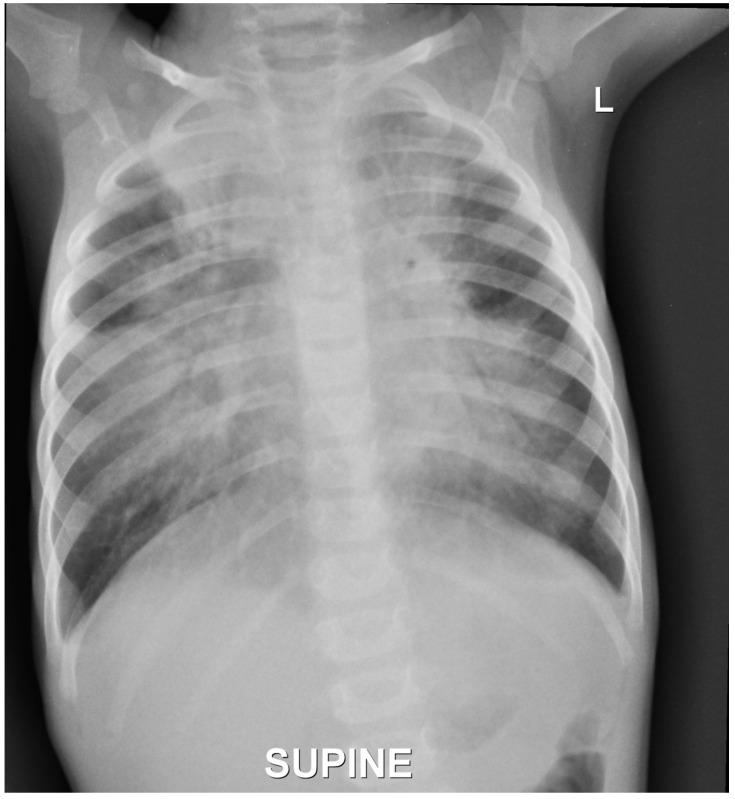
Chest X-ray showing extensive bronchopneumonia.

The child was treated with intravenous antibiotics and antiviral medications along with oxygen driven nebulisations and intravenous fluids. She was also given proton pump inhibitors intravenously. Her blood investigations showed raised C-reactive protein (CRP) with neutrophilic leukocytosis and the blood cultures were negative. The chest X-ray showed extensive bronchopneumonia (Figure 1). A repeat cardiac echo and a thyroid screen were done which was normal. She improved symptomatically with therapy, but began having emesis again when oral feeds were resumed with cereals.

**Figure 2:**
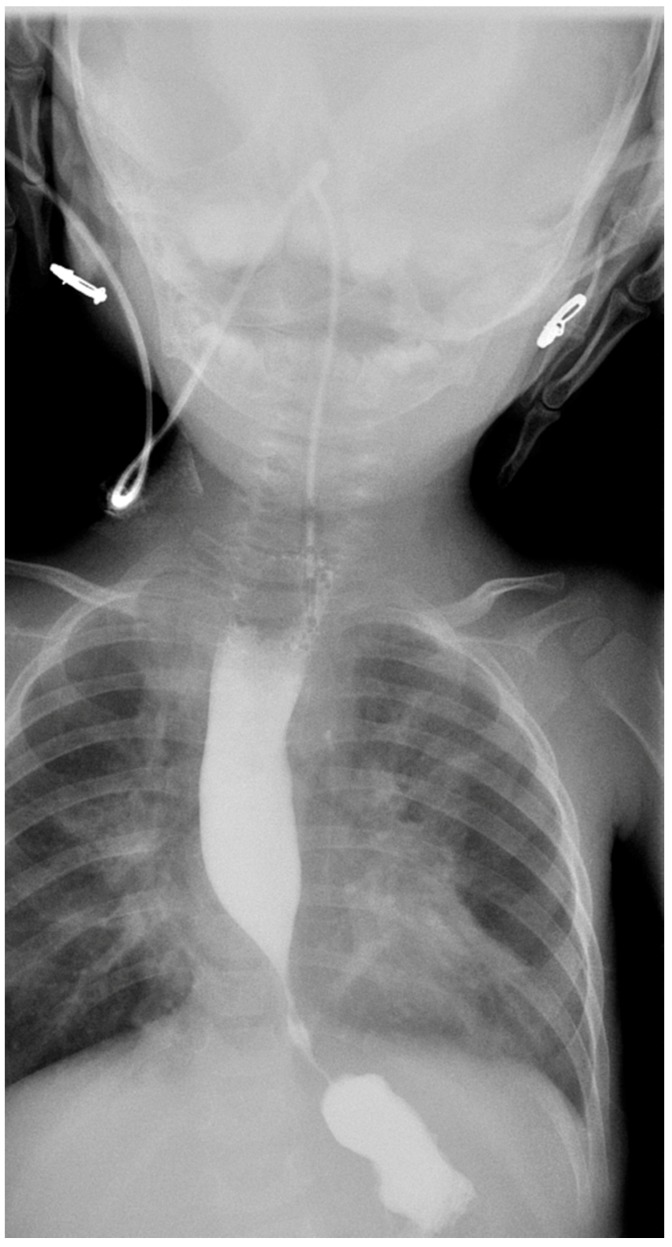
Barium swallow showing an obstruction at the lower end of the esophagus with proximal dilatation and pooling of barium.

A gastroenterology consultation was taken in view of the recurrent vomiting and aspiration pneumonia with failure to thrive. A barium swallow (Figure 2) was done, which showed an obstruction at the lower end of the esophagus with proximal dilatation and pooling of barium.

**Figure 3:**
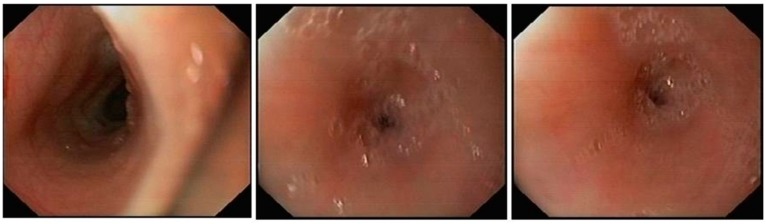
Endoscopy showing an esophageal web with a pinpoint orifice.

She was fed exclusively via an orogastric tube for a few days to prevent further episodes of aspiration and to help her lungs clear up in readiness for anesthesia. Her pneumonia improved and she was taken up for an endoscopic evaluation under general anesthesia. On endoscopy (Figure 3), an esophageal web of thickness less than 5 mm with a pinpoint orifice was noted in the lower esophagus just above the gastroesophageal junction, and was dilated using a controlled radial expansion balloon from 8 to 12 mm. There was only mucosal and sub mucosal involvement with no involvement of the muscular portion of the esophagus. There were no features of esophagitis or endoscopic findings suggestive of achalasia.

Initially, she was kept nil per oral and then fed liquids via the nasogastric tube, and then semisolids orally. Post procedure, she had some feeding issues with intermittent vomiting, but she improved and at the time of discharge, she was on full oral feeds with no vomiting or further signs of aspiration. She was discharged with a weight of 6.35 kg (an increase of 1.35 kg), length of 68 cm, and a head circumference of 42 cm. At eight months following the endoscopy, she continues to be well and is thriving.

## 3. Discussion

Down syndrome can occur in three different genetic variations. Trisomy 21 is the most common form [3]. Translocation causes 3% of the total cases, where extra chromosomes are attached or translocated to a different chromosome [3]. Mosaicism results in 2% of cases, where a few cells contain two chromosomes and a few with extra chromosome [3].

Gastrointestinal symptoms are common in Down syndrome, both in children and adults. It includes vomiting, diarrhea, constipation and abdominal pain. These symptoms often resolve spontaneously. However, structural and functional disorders of the gastrointestinal tract are more common in Down syndrome [4].

Structural defects of gastrointestinal tract in Down syndrome include esophageal, duodenal, and small bowel atresia or stenosis, annular pancreas, causing small bowel obstruction, imperforate anus, and Hirschsprung disease [5]. Gastrointestinal obstruction may be detected before birth by imaging techniques, so that interventions can be planned early after birth. If diagnosis is not made pre-birth, symptoms of vomiting, absent bowel sounds in a distressed baby, indicating that abdominal pain should evoke suspicion of bowel obstruction and the need for emergency surgical intervention.

In children who appear uncomfortable during or after feeding, gastroesophageal reflux should be suspected. Down syndrome children spend less time in sitting position. Moreover, their lower esophageal muscle sphincter tone is reduced, thus allowing for gastroesophageal reflux [6,7]. This usually presents as aspiration pneumonia and early evaluation of esophageal function should be done in children with chronic cough or recurrent pneumonia. Gastroesophageal reflux is often misdiagnosed as asthma and remains untreated.

Duodenal atresia is said to be the most common defect that presents with severe vomiting from birth. This is a rare case report of Down syndrome with esophageal web that presented with recurrent vomiting and aspiration since birth.

Esophageal webs are more commonly found in females, but multiple rings or webs are common in males. They can be Congenital or Acquired. Acquired causes include gastroesophageal reflux disease, Plummer Vinson syndrome, iron deficiency anemia, celiac sprue, inlet patch, graft versus host disease, and skin diseases, drug induced, mediastinal radiation, and corrosive ingestion [8].

Esophageal webs are classified as type A, B and C, the most common being the type B ring, otherwise called Schatzki’s ring located at the squamocolumnar junction [9,10]. Various theories have been proposed in the formation of esophageal ring. Congenital theory states that esophageal web is a result of failure in the complete recanalization of esophagus. Gastric acid symptoms are the main presentation of those with Schatzki’s ring. Recurrence can be prevented by acid suppression following dilatation.

In our case, due to the pin point orifice of the web, ingested food was accumulating in the dilated proximal segment with a spill over into the respiratory tract leading to recurrent aspiration pneumonia. The web was successfully treated with a single endoscopic dilatation without recurrence of symptoms as of last follow up eight months post-procedure.
